# Legume Lectins with Different Specificities as Potential Glycan Probes for Pathogenic Enveloped Viruses

**DOI:** 10.3390/cells11030339

**Published:** 2022-01-20

**Authors:** Annick Barre, Els J. M. Van Damme, Bernard Klonjkowski, Mathias Simplicien, Jan Sudor, Hervé Benoist, Pierre Rougé

**Affiliations:** 1 UMR 152 PharmaDev, Institut de Recherche et Développement, Faculté de Pharmacie, Université Paul Sabatier, 35 Chemin des Maraîchers, F-31062 Toulouse, France; annick.barre@univ-tlse3.fr (A.B.); simplicien.mathias@gmail.com (M.S.); jan.sudor1@univ-tlse3.fr (J.S.); herve.benoist@ird.fr (H.B.); 2 Department of Biotechnology, Faculty of Bioscience Engineering, Ghent University, Coupure Links 653, B-9000 Ghent, Belgium; elsjm.vandamme@ugent.be; 3 UMR Virologie, INRA, ANSES, Ecole Nationale Vétérinaire d’Alfort, F-94700 Maisons-Alfort, France; bernard.klonjkowski@vet-alfort.fr

**Keywords:** enveloped virus, Ebola virus, HIV, herpes simplex virus, human cytomegalovirus, influenza virus, MERS-CoV, SARS-CoV-2, *N*-glycosite, *O*-glycosite, high-mannose glycan, complex *N*-glycans, Vicieae man-specific lectin, T/Tn-specific lectin, specific interaction

## Abstract

Pathogenic enveloped viruses are covered with a glycan shield that provides a dual function: the glycan structures contribute to virus protection as well as host cell recognition. The three classical types of *N*-glycans, in particular complex glycans, high-mannose glycans, and hybrid glycans, together with some *O*-glycans, participate in the glycan shield of the Ebola virus, influenza virus, human cytomegalovirus, herpes virus, human immunodeficiency virus, Lassa virus, and MERS-CoV, SARS-CoV, and SARS-CoV-2, which are responsible for respiratory syndromes. The glycans are linked to glycoproteins that occur as metastable prefusion glycoproteins on the surface of infectious virions such as gp120 of HIV, hemagglutinin of influenza, or spike proteins of beta-coronaviruses. Plant lectins with different carbohydrate-binding specificities and, especially, mannose-specific lectins from the Vicieae tribe, such as pea lectin and lentil lectin, can be used as glycan probes for targeting the glycan shield because of their specific interaction with the α1,6-fucosylated core Man_3_GlcNAc_2_, which predominantly occurs in complex and hybrid glycans. Other plant lectins with Neu5Ac specificity or GalNAc/T/Tn specificity can also serve as potential glycan probes for the often sialylated complex glycans and truncated *O*-glycans, respectively, which are abundantly distributed in the glycan shield of enveloped viruses. The biomedical and therapeutical potential of plant lectins as antiviral drugs is discussed.

## 1. Introduction

Many pathogenic viruses for humans are so-called enveloped viruses with a lipid bilayer that allows the infectious virions to fuse with the cell membrane, followed by the entry and replication of the viral genetic material into the host cells. Ebola virus (EBOV), influenza virus (IV), herpes simplex virus (HSV), human immunodeficiency virus (HIV), human cytomegalovirus (HCMV), Lassa virus (LASV), and the beta-coronaviruses responsible for the Middle East respiratory syndrome (MERS-CoV) and the severe acute respiratory syndrome (i.e., SARS-CoV and SARS-CoV-2) belong to this group of pathogenic enveloped viruses [1]. Among the surface glycoproteins that are embedded in the lipid bilayer of enveloped viruses, so-called fusion glycoproteins play a key role in mediating the recognition of infectious virions by the host cell membrane receptors and their subsequent anchorage to the host cells [2]. Energetically driven conformational changes occurring in the metastable fusion proteins, which usually occur in a prefusion state, are responsible for an enhanced exposure of the receptor-binding domain (RBD) of the fusion proteins that favors their recognition by the host cell receptors [1]. Fusion proteins of enveloped viruses usually consist of the non-covalent association of three monomers to build a homotrimeric structure exposed on the surface of the virions. However, depending on the enveloped viruses, the structure, shape, and size of the monomers building the homotrimers are highly variable from one virus to another (Table 1). Other surface glycoproteins, such as the so-called B glycoprotein from HSV [3], and E proteins from flaviviruses responsible for some severe diseases, including chikungunya virus (CHIV), dengue virus (DENV), and Zika virus (ZIV), also contribute to the glycan shield covering the infectious virions [4,5,6].

**Table 1:** Structural properties of fusion protein and E glycoprotein associations of enveloped viruses as parts of the glycan shield covering infectious virions.

Enveloped Virus	Homotrimer	Monomer	PDB Entry *	Reference
Ebola virus (EBOV)	Homotrimer		7JPH	[7]
Influenza virus (IV)	Hemagglutinin A Homotrimer	hemagglutinin A	6Y5G	[8]
Human cytomegalovirus (HCMV)	Homotrimer	B glycoprotein	5CXF	[9]
Herpes simplex virus (HSV)	Homotrimer	B glycoprotein	2GUM	[3]
Human immunodeficiency virus (HIV)	Homotrimer	gp140	4TVP	[10]
Lassa virus (LASV)	Homotrimer	GPC glycoprotein	5VK2	[11]
Middle east respiratory syndrome coronavirus (MERS-CoV)	Spike	S protein	5W9H	[12]
Severe acute respiratory syndrome coronavirus-1 (SAR-CoV)	Spike	S protein	6ACD	[13]
Severe acute respiratory syndrome coronavirus-2 (SARS-CoV-2)	Spike	S protein	6VXX	[14]
Chikungunya virus (CHIV)	Homodimer	E protein	3N40	[4]
Dengue virus (DENV)	Homodimer	E protein	1UZG	[5]
Zika virus (ZIV)	Homodimer	E protein	57BUB	[6]

* A single PDB code is indicated but several PDB entries are available at the PDB.

Although *N*- and *O*-glycans decorate the fusion glycoproteins, the three classical types of *N*-glycans, including complex-type glycans, high-mannose-type glycans, and hybrid-type glycans, are predominantly distributed along the fusion proteins of pathogenic enveloped viruses. In addition, a high proportion of complex glycans are α1,6-fucosylated on the first GlcNAc linked to the Asn residue and often sialylated on their terminal Gal residues [15]. Moreover, the extreme diversity of complex glycans appears as a characteristic of enveloped viruses. In addition to the *N*-glycans, *O*-glycans have been identified on the envelope glycoproteins of infectious virions, especially in pathogenic coronaviruses such as SARS-CoV-2 [16]. In fact, most of the Ser and Thr residues of unoccupied NXT/S glycosylation sites of SARS-CoV-2 are *O*-glycosylated by short Gal/GalNAc/T/Tn-containing *O*-glycan chains [17]. However, the *O*-glycan content of SARS-CoV-2 is much lower than the level of *N*-glycans.

Lectins are known as a group of carbohydrate-binding proteins of non-immune origin that are widely distributed in plants. Many lectins have been studied for their role in the protection of plants against pathogens, aiming to resolve the function of the lectins inside different plant tissues. In addition, these carbohydrate-binding proteins have been proven to be important tools for glycobiology, allowing for the investigation of the importance of protein–carbohydrate interactions. Several plant lectins have been reported as potent molecules with anti-infectivity properties for RNA viruses including pathogenic enveloped viruses. Depending on their carbohydrate-binding specificity, lectins can recognize and bind particular types of glycan structures present in the glycan shield of viruses. Over the past decades, lectins from different legume species, referred to as legume lectins, have been studied in great detail. Despite the fact that legume lectins represent a large family of proteins with important similarity in their amino acid sequences, these lectins show remarkable variability in their carbohydrate-binding properties. Many legume lectins have been reported to recognize glycoconjugates on cells and viruses and can discriminate between diverse glycan structures, making them interesting research tools for glycomic research [18].

The primary purpose of this review was to give an overview of the types of glycans present in the glycan shield of different pathogenic enveloped viruses and how legume lectins with different specificities can act as carbohydrate-binding agents (CBAs) for these viruses. Finally, biomedical perspectives for plant lectins with antiviral properties are also discussed.

## 2. The Glycan Shield of Pathogenic Enveloped Viruses

**Figure 1:**
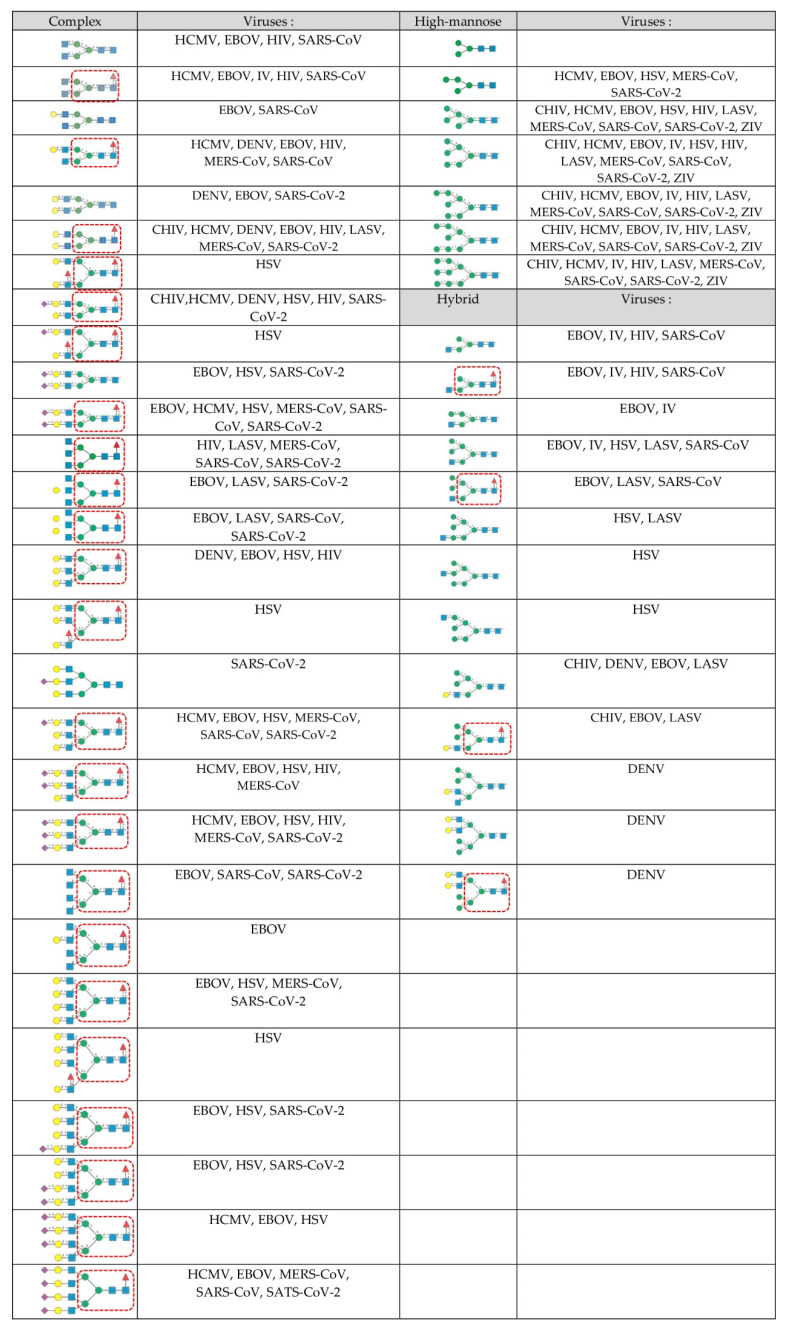
Diversity of the different types of *N*-glycans forming the glycan shield covering the pathogenic enveloped viruses: Ebola virus (EBOV) [19], herpes simplex virus (HSV) [20], human cytomegalovirus (HCMV) [21], human immunodeficiency virus (HIV) [22], influenza virus (IV) [23], chikungunya virus (CHIV) [24], Lassa virus (LASV) [25], MERS-CoV (MERS-CoV) [15], SARS-CoV (SARS-CoV) [15], SARS-CoV-2 (SARS-CoV-2) [15], and Zika virus (ZIV) [26]. Symbols representing the glycan structures are as follows: GlcNAc (blue square), Gal (yellow circle), Man (green circle), Fuc (red triangle), and Neu5Ac (purple diamond).

Glycoproteins that are part of the glycan shield that covers the enveloped viruses are modified with three types of *N*-glycans including complex glycans, high-mannose glycans, and hybrid glycans (Figure 1):

- Complex-type *N*-glycans are most abundant on all the envelope proteins except for the gp120 and hemagglutinin from HIV and IV, respectively, which predominantly contain high-mannose-type *N*-glycans. Complex glycans exhibit a high diversity in their glycan structure, including bi-, tri-, and tetra-antennary glycans which are often sialylated on their terminal Gal residues and fucosylated on sub-terminal GlcNAc residues. Most of these complex *N*-glycans possess an α1,6-fucosylated Man_3_GlcNAc_2_ core;
- High-mannose *N*-glycans are less abundant and offer less diversity than complex *N*-glycans because they consist exclusively of Man residues. High-mannose *N*-glycans from enveloped viruses include oligomannosides containing 4–9 (Man_4–9_) Man residues, and all of them possess a non-fucosylated Man_3_GlcNAc_2_ core;
- Hybrid *N*-glycans are least abundant on enveloped viruses.

A detailed study of the *N*-glycan structures occurring on the beta-coronaviruses, MERS-CoV, SARS-CoV, and SARS-CoV-2, confirmed the high heterogeneity of the complex glycans of the S glycoprotein forming the spikes and revealed important differences depending on the type of beta-coronavirus [15]. In addition, although some of the *N*-glycosylation sites, NXT/S, are often occupied with variable proportions of complex and high-mannose *N*-glycans, the complex *N*-glycans are largely predominant [16,27,28,29].

Furthermore, a few *O*-glycans also occur, especially on the S protein from beta-coronaviruses [16]. Interestingly, the Thr and Ser residues of *N*-glycosylation sites unoccupied by *N*-glycans are modified with short *O*-glycan chains [17]. Usually, these *O*-glycans are less exposed on the surface of S proteins, mainly due to the fact of their smaller size compared to the large and highly exposed *N*-glycans [27].

**Figure 2:**
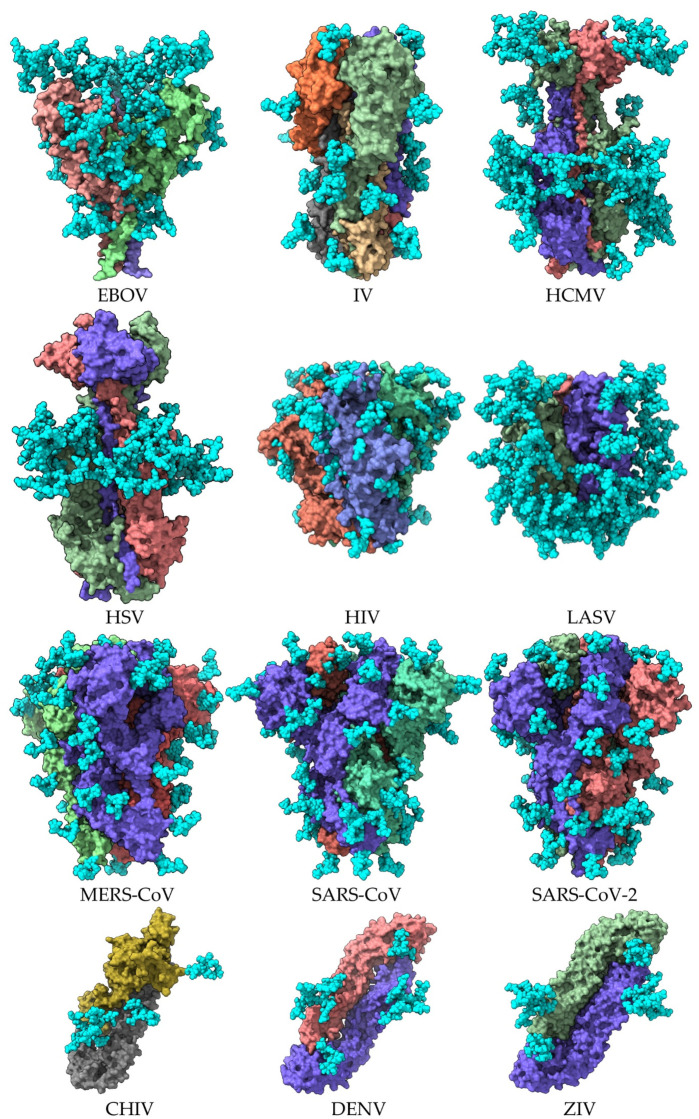
Figure illustrating the extent of the glycan shield covering the envelope glycoproteins of pathogenic enveloped viruses. The illustrations show the lateral face of the molecular surface of the homotrimeric organization of the envelope glycoprotein of Ebola virus (EBOV) (PDB code 7JPH), influenza virus (IV) (PDB code 6Y5G), human cytomegalovirus (HCMV) (PDB code 5CXF), herpes simplex virus (HSV) (PDB code 2GUM), human immunodeficiency virus HIV (PDB code 4TVP), Lassa virus (LASV) (PDB code 5VK2), Middle East respiratory syndrome virus (MERS-CoV) (PDB code 5W9H), severe acute respiratory syndrome (SARS-CoV) (PDB code 6ACD), and severe acute respiratory syndrome-2 (SARS-CoV-2) (PDB code 6VXX). Lateral face of the molecular surface of the dimeric organization of the E glycoprotein of chikungunya virus CHIV (PDB code 3N40), dengue virus DENV (PDB code 1UZG), and Zika virus ZIV (PDB code 7BUB). Monomers forming the homotrimeric and homodimeric associations of envelope glycoproteins are colored differently, and *N*-glycan chains forming the glycan shield are represented by cyan colored balls.

**Figure 3:**
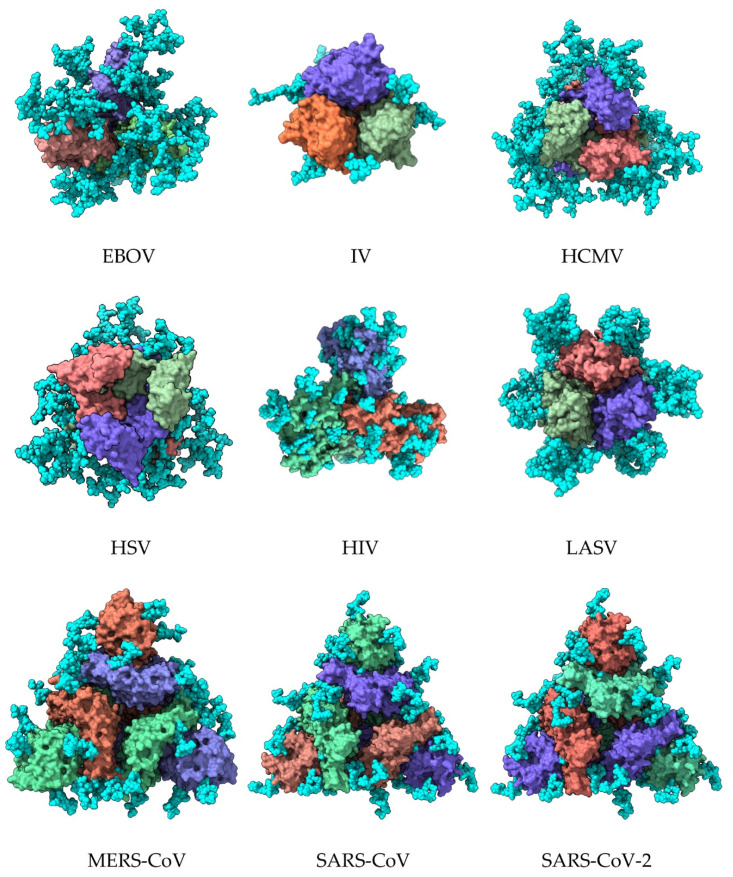
Figure illustrating the extent of the glycan shield covering the envelope glycoproteins of pathogenic enveloped viruses. Illustrations show the top face of the molecular surface of the homotrimeric organization of the envelope glycoprotein of Ebola virus (EBOV) (PDB code), influenza virus (IV) (PDB code), human cytomegalovirus (HCMV) (PDB code 5CXF), herpes simplex virus (HSV) (PDB code 2GUM), human immunodeficiency virus (HIV) (PDB code 4TVP), Lassa virus (LASV) (PDB code 5VK2), Middle East respiratory syndrome virus (MERS-CoV) (PDB code 5W9H), severe acute respiratory syndrome (SARS-CoV) (PDB code 6ACD), and severe acute respiratory syndrome-2 (SARS-CoV-2) (PDB code 6VXX). Monomers forming the homotrimeric associations of envelope glycoproteins are colored differently, and *N*-glycan chains forming the glycan shield are represented by cyan colored balls.

Both the homotrimeric organization of the fusion proteins and the homodimeric organization of E glycoproteins on the surface of pathogenic enveloped viruses favor the exposure of their glycan shield (Figure 2 and Figure 3). However, the distribution of *N*-glycans, especially at the top of the fusion protein homotrimer, provides areas devoid of glycans allowing for the recognition of pathogenic viruses by the corresponding DPP4 and ACE2 receptors located on the host cells. These glycan-free areas, which correspond to the so-called RBDs of S proteins from MERS-CoV, SARS-CoV, and SARS-CoV-2, contribute to the infectious potential developed by the pathogenic beta-coronaviruses [28,29,30].

In spite of the glycan-free character of the RBDs, it should be noted that these areas are surrounded by glycan chains that should be accessible to CBAs, such as lectins, which could hamper the proper recognition of RBDs by their corresponding host cell receptors (Figure 3) [14].

## 3. Plant Lectins with Different Specificities Are Potential CBAs for Pathogenic Enveloped Viruses

**Figure 4:**
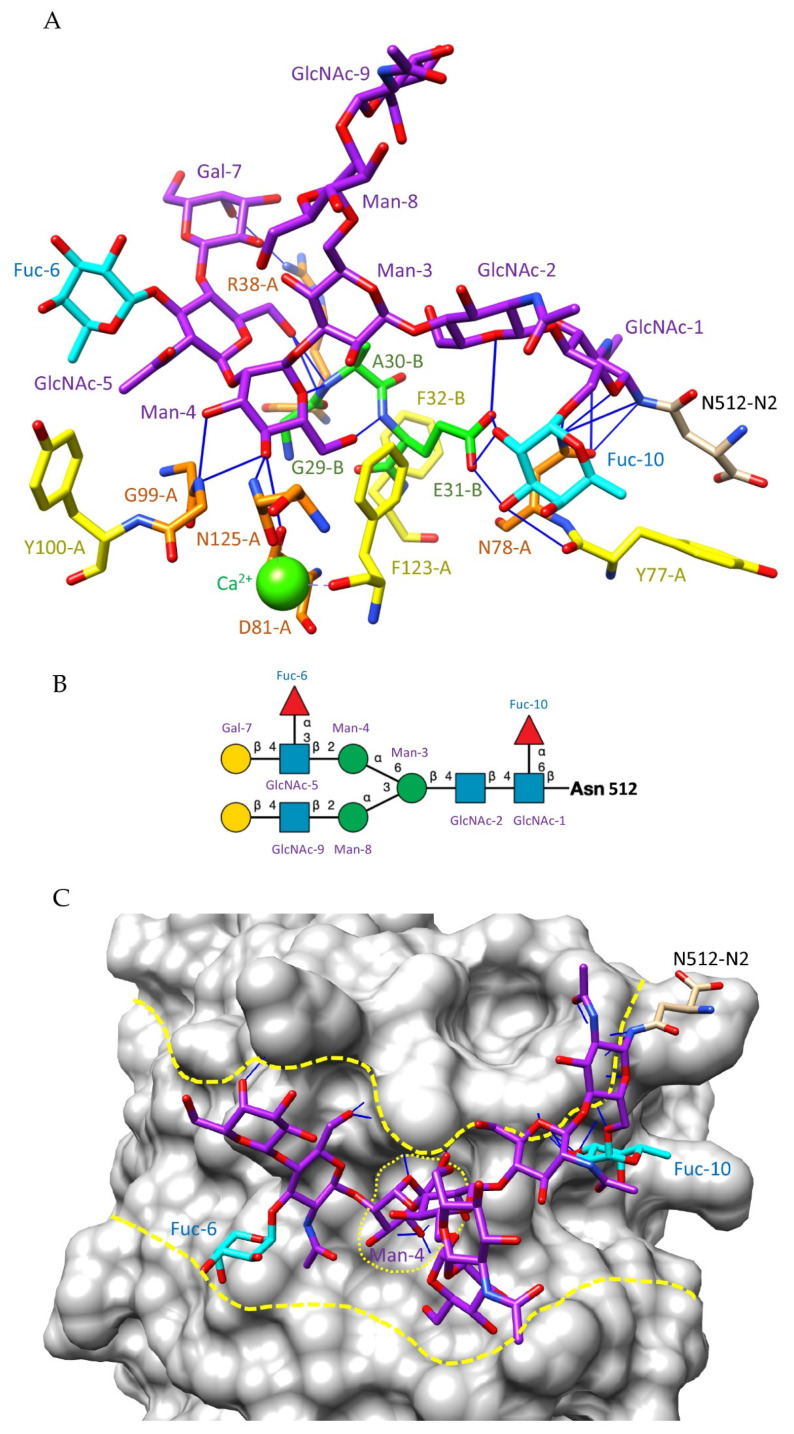
Figure illustrating the interaction of the Man-specific lectin LoL-II from *Lathyrus ochrus* with an oligosaccharide chain. (**A**) Network of hydrogen bonds (black lines) anchoring N2 oligosaccharide (colored purple) to LoL-II isolectin from *Lathyrus ochrus* (PDB code 1LGC). Hydrophilic residues R38, N78, D81, G99, and N125 of the α-chain and E31 of the β-chain, which participate in hydrogen bonds, are colored orange and green, respectively. Aromatic residues Y77, Y100, F123, Y124, and W128 of the α-chain and F32 of the β-chain, involved in stacking interactions with the pyranose rings of the oligosaccharide, are colored yellow. The α1,6-linked fucose (Fuc), which participates in the H-bond network, is colored cyan. (**B**) Depiction of the N2 oligosaccharide using the symbol nomenclature for glycans: Fuc (red triangle), Gal (yellow circle), GalNAc (yellow square), GlcNAc (blue square), Man (green circle), and sialic acid/Neu5Ac (purple diamond). (**C**) Molecular surface of the N2 oligosaccharide–Lo-LII complex, showing how the isolectin accommodates the oligosaccharide via a network of hydrogen bonds and stacking interactions. The groove harboring the N2 oligosaccharide and the central monosaccharide-binding site of the lectin are delineated with yellow, dashed lines.

Lectins from higher plants offer extreme diversity in terms of structural organization and recognition of simple and complex glycans [31]. Owing to the high diversity that characterizes the glycan shield of pathogenic enveloped viruses, the heterogeneous group of Man-specific lectins and, especially, the group of two-chain legume lectins, emerges as a potential tool for specific targeting of the *N*-glycan shield of enveloped viruses. Two-chain legume lectins form a particular group of Man-specific lectins that display an enhanced affinity for complex *N*-glycans possessing an α1,6-fucosylated trimannoside core Man_3_GlcNAc_2_ [32,33]. Seed lectins from pea (*Pisum sativum*) (PsA), lentil (*Lens culinaris*) (LcA), Cyprus-vetch (*Lathyrus ochrus*) (LoL-I/II), and faba bean (*Vicia faba*) (VfA) belong to this group of two-chain lectins [34,35,36,37]. They are built from the non-covalent association of two identical monomers built up from a heavy (β-chain) and a light (α-chain) subunit and possess an overall jelly roll structure similar to that of Con A, the single-chain Man-binding lectin from Jackbean (*Canavalia ensiformis*) [38]. A detailed crystallographic study of the *Lathyrus ochrus* isolectin-II (LoL-II) in complex with an octasaccharide derived from the human lactotransferrin (PDB code 1LGC) [39] revealed that the enhanced affinity of Vicieae lectins towards the α1,6-fucosylated Man3GlcNAc2 core depends on the direct interaction of the α1,6-linked Fuc residue with some of the amino acid residues forming the carbohydrate-binding site (CBS) of the lectin via a few hydrogen bonds (Figure 4).

**Figure 5:**
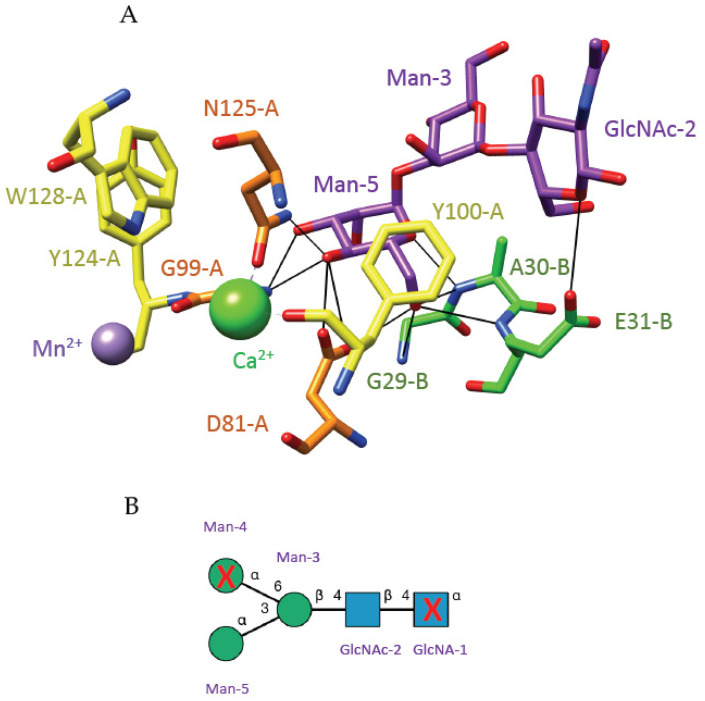
Figure illustrating the interaction of the Man-specific lectin LoL-I from *Lathyrus ochrus* with a trimannoside. (**A**) Network of hydrogen bonds (black lines) anchoring the trisaccharide Manα1,3Manβ1,4GlcNAc (colored purple) to LoL-I isolectin from *Lathyrus ochrus* (PDB code 1LOG). Hydrophilic residues D81, G99, and N125 of the α-chain and G29, A30, and E31 of the β-chain, which participate in hydrogen bonds, are colored orange and green, respectively. Aromatic residues Y100, Y124, and W128 of the α-chain, involved in stacking interactions with the pyranose rings of the oligosaccharide, are colored yellow. (**B**) Illustration of the Man_3_GlcNAc_2_ oligosaccharide using the symbol nomenclature for glycans: Fuc (red triangle), Gal (yellow circle), GalNAc (yellow square), GlcNAc (blue square), Man (green circle), and sialic acid/Neu5Ac (purple diamond), showing the sugar units that participate in the trisaccharide–LoL-I complex.

In addition, complexes of pea lectin and *Lathyrus ochrus* lectin with non-fucosylated trisaccharides, indicated that Vicieae lectins also interact with one of the terminal Man of the trimannosyl core from the non-fucosylated *N*-glycans [40]. In this respect, LoL-I from *L. ochrus* seeds interacted with the trisaccharide Manα1,3Manβ1,4GlcNAc in such a way that the terminal Man residue occupies the monosaccharide-binding site of the lectin (Figure 5).

This binding pattern allows Vicieae lectins to readily interact with the trimannosyl core from the three types of complex, high-mannose, and hybrid *N*-glycans, irrespective of the possible α1,6-fucosylation on the first GlcNAc residue of the *N*-glycan chain.

**Figure 6:**
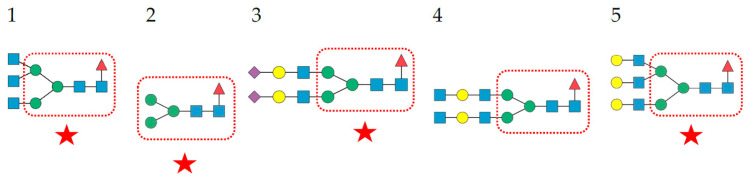
Structures of *N*-glycans recognized by PsA. Top 5 *N*-glycans are arranged in decreasing order of affinity for PsA. The α1,6-fucosylated Man_3_GlcNAc_2_ core is delineated with a red square. Glycans occurring in the glycan shield of pathogenic enveloped viruses are indicated by a red star. Fuc (red triangle), Gal (yellow circle), GalNAc (yellow circle), GlcNAc (blue square), Man (green circle), and sialic acid/Neu5Ac (purple diamond).

A survey of the glycan array analyses performed by the Consortium for Functional Glycomics (CFG) (http://www.functionalglycomics.org (accessed on 15 December 2021)) for PsA, LcA, and VfA, all members of the two-chain lectins from the Vicieae tribe, yielded the best results with glycans possessing the a1,6-fucosylated Man_3_GlcNAc_2_ core. As an example, most of the top five glycans displaying the best affinity for PsA, LcA, and VfA occur in the glycan shield covering the pathogenic enveloped viruses (Figure 6).

**Figure 7:**
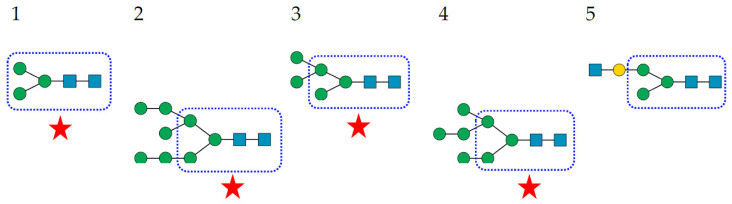
Structures of *N*-glycans recognized by GNA. The top 5 *N*-glycans are arranged in decreasing order of affinity for GNA. The Man_3_GlcNAc_2_ core is delineated by a blue square. High-mannose glycans occurring in the glycan shield of pathogenic enveloped viruses are indicated by a red star. Gal (yellow circle), GalNAc (yellow circle), GlcNAc (blue square), Man (green circle).

Other Man-specific lectins, such as the GNA-related lectins from different families of monocot plants, including Liliaceae, Amaryllidaceae, Polygonaceae, and Orchidaceae, preferentially interact with high-mannose glycans that contain a non-fucosylated Man_3_GlcNAc_2_ core. As an example, the top five glycans interacting with GNA in glycan array experiments mainly consist of high-mannose glycans (Figure 7). These high-mannose glycans were present in all the investigated enveloped viruses.

**Figure 8:**
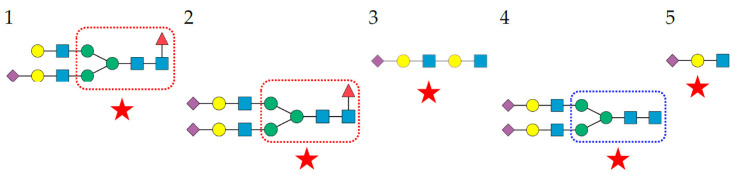
Structures of *N*-glycans recognized by SNA-I. The top five sialylated *N*-glycans are arranged in decreasing order of affinity for SNA-I. The Man_3_GlcNAc_2_ core is delineated by a blue square. The fucosylated Man_3_GlcNAc_2_ core is indicated with a red circled. Sialylated glycans occurring in the glycan shield of pathogenic enveloped viruses are indicated by a red star. Fuc (red triangle), Gal (yellow circle), GalNAc (yellow circle, GlcNAc (blue square), Man (green circle), and sialic acid/Neu5Ac (purple diamond).

In addition, the *N*-glycans of pathogenic enveloped viruses are often sialylated on their terminal Gal antennae residues, which offers another potential recognition target for lectins that specifically recognize terminal sialylated Gal residues. The black elderberry (*Sambucus nigra*) bark lectin I (SNA-I) specifically interacts with these sialylated termini. In this respect, the top five glycans interacting with SNA-I in glycan array experiments contained sialylated Gal residues that occur in the glycan shield of enveloped viruses (Figure 8).

**Figure 9:**
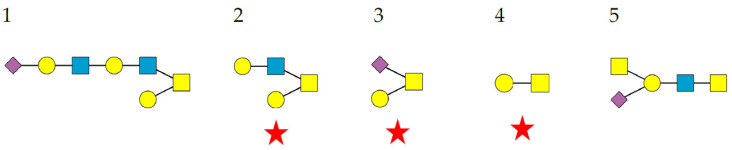
Structures of *O*-glycans recognized by Morniga-G. The top 5 *O*-glycans are arranged in decreasing order of affinity for PNA. The *O*-glycans occurring in the glycan shield of SARS-CoV-2 spike protein are indicated by a red star. Gal (yellow circle), GalNAc (yellow square), GlcNAc (blue square), and sialic acid/Neu5Ac (purple diamond).

Finally, GalNAc/T/Tn lectins that recognize O-glycans, such as jacalin from *Artocarpus integer*, PNA from peanut (*Arachis hypogaea*), and Morniga-G from *Morus nigra*, should especially interact with the few *O*-glycans exposed at the surface of beta-coronaviruses [41]. The top five *O*-glycans interacting with Morniga-G in glycan array experiments are present in the glycan shield of the SARS-CoV-2 particles (Figure 9).

## 4. Man-Specific and Neu5Ac-Specific Lectins as Potential CBAs for Pathogenic Enveloped Viruses

Plant lectins with different carbohydrate-binding specificities have been identified as CBAs for pathogenic enveloped virus including HIV, HCMV, the hepatitis C virus HCV, HSV, IV, and the coronaviruses MERS-CoV, SARS-CoV, and SARS-CoV-2. Targets for these CBAs are the glycan structures present on the envelope proteins of pathogenic enveloped viruses (Table 2).

**Table 2:** List of plant lectins identified as carbohydrate-binding agents (CBAs) for envelope proteins from pathogenic enveloped viruses.

Lectin	Plant Species	Carbohydrate- Binding Specificity	Targeted Envelope Protein	Virus	Ref.
APA	*Allium porum*	Man	S-protein	SARS-CoV	[41]
AUA	*Allium ursinum*	Man	S-protein	SARS-CoV	[41]
BanLec	*Musa acuminata*	Man	gp120	HIV	[42,43,44]
			hemagglutinin	IV	[45]
			E-glycoprotein	HCMV	[46]
			E-glycoprotein	EBOV	[46,47]
			E-glycoprotein	LASV	[46]
Con A	*Canavalia ensiformis*	Man	gp120	HIV	[48]
			S-protein	SARS-CoV-2	[49]
Succinyl-Con A	*Canavalia ensiformis*	Man	S-protein	Mers-CoV	[50]
			S-protein	SARS-CoV	[50]
			S-protein	SARS-CoV-2	[50]
ConBr	*Canavalia brasiliensis*	Man	S-protein	SARS-CoV-2	[51]
ConM	*Canavalia maritima*	Man	S-protein	SARS-CoV-2	[51]
CLA	*Cladastris lutea*	Man	S-protein	SARS-CoV	[41]
CHA	*Cymbidium* hybrid	Man	β-glycoprotein	HCMV	[52]
			E-glycoprotein	HCV	[52]
			gp120	HIV	[52]
			S-protein	SARS-CoV	[53]
DSL	*Datura stramonium*	Neu5Ac-Gal/GalNAc	S-protein	MERS-CoV	[50]
			S-protein	SARS-CoV	[50]
			S-protein	SARS-CoV-2	[50]
DLasL	*Dioclea lasiocarpa*	Man	S-protein	SARS-CoV-2	[51]
DSclerL	*Dioclea sclerocarpa*	Man	S-protein	SARS-CoV-2	[51]
EHA	*Epipactis helleborine*	Man	β-glycoprotein	HCMV	[52]
			gp120	HIV	[52]
			hemagglutinin	IV	[52]
GNA	*Galanthus nivalis*	Man	β-glycoprotein	HCMV	[53]
			E-glycoprotein	HCV	[54,55]
			gp120	HIV	[53]
			S-protein	SARS-CoV	[41]
			S-protein	SARS-CoV-2	[56]
			hemagglutinin	IV	[57]
HHA	*Hyppeastrum* hybrid	Man	β-glycoprotein	HCMV	[53]
			E-glycoprotein	HCV	[52]
			gp120	HIV	[52]
			S-protein	SARS-CoV	[41]
			hemagglutinin	IV	[57]
Horcolin	*Hordeum vulgare*	Man	gp120	HIV	[58]
IRA	*Iris* hybrid	GalNAc/Gal	S-protein	SARS-CoV	[41]
FRIL	*Lablab purpureus*	Man	S-protein	SARS-CoV	[59]
			hemagglutinin	IV	[59]
LcA	*Lens culinaris*	Man	S-protein	MERS-CoV	[50]
			S-protein	SARS-CoV	[50]
			S-protein	SARS-CoV-2	[50]
LOA	*Listera ovata*	Man	β-glycoprotein	HCMV	[53]
			gp120	HIV	[53]
MAL	*Maackia amurensis*	Neu5Ac	S-protein	SARS-CoV-2	[60]
Morniga-G	*Morus nigra*	Gal	S-protein	SARS-CoV	[41]
Morniga-M	*Morus nigra*	Man	S-protein	SARS-CoV	[41]
NPA	*Narcissus pseudonarcissus*	Man	β-glycoprotein	HCMV	[53]
			gp120	HIV	[53]
Nictaba	*Nicotiana tabacum*	(GlcNAc)n	S-protein	SARS-CoV	[41,61]
Orysata	*Oryza sativa*	Man	gp120	HIV	[62]
			S-protein	SARS-CoV	[62]
PHA	*Phaseolus vulgaris*	Complex glycans	S-protein	MERS-CoV	[50]
			S-protein	SARS-CoV	[50]
			S-protein	SARS-CoV-2	[50]
PCA	*Polygonatum cyrtonema*	Man	gp120	HIV	[63]
SSL	*Sambucus sieboldiana*	Neu5Ac-Gal/GalNAc	S-protein	SARS-CoV	[50]
			S-protein	SARS-CoV-2	[50]
TLC II	*Tulipa* hybrid	Man	S-protein	SARS-CoV	[41]
TDL	*Typhonium divaricatum*	Man	E-glycoprotein	HSV	[64]
UDA	*Urtica dioica*	(GlcNAc)n	β-glycoprotein	HCMV	[52]
			E-glycoprotein	HCV	[51]
			gp120	HIV	[52]
			S-protein	SARS-CoV	[41]
			hemagglutinin	IV	[57]
ML II	*Vicum album*	Gal/GalNAc	S-protein	SARS-CoV	[41]
ML III	*Viscum album*	Gal/GalNAc	S-protein	SARS-CoV	[41]
WGA	*Triticum aestivum*	GlcNAc/Neu5Ac	S-protein	MERS-CoV	[50]
			S-protein	SARS-CoV	[41,50]
			S-protein	SARS-CoV-2	[50,65]
GNAmaize	*Zea mays*	Man	S-protein	SARS-CoV	[66]

**Figure 10:**
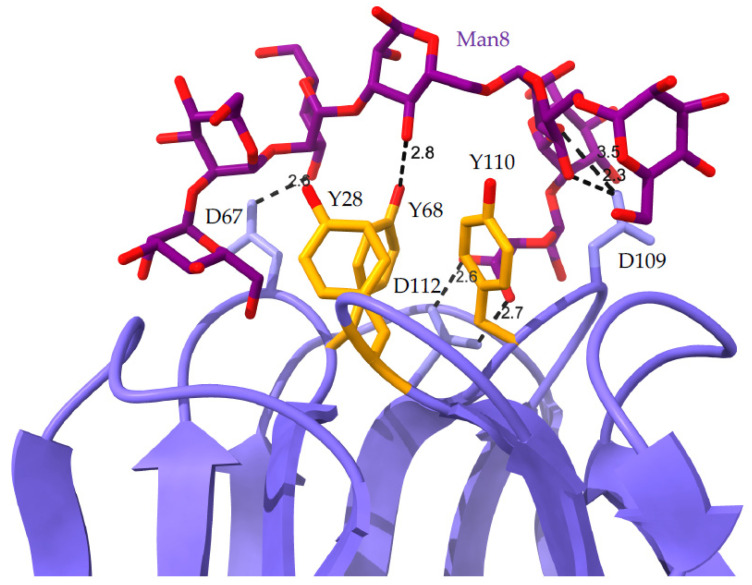
Figure illustrating how the Man-specific lectin griffithsin interacts with an oligomannoside. Network of hydrogen bonds (black, dashed lines) connecting the griffithsin monomer (colored violet) to a linear Man8 chain (colored purple) (PDB code 3LL2). Amino acid D residues participating in hydrogen bonds are labeled (i.e., D67, D109, and D112). Aromatic Y residues involved in stacking interactions with the sugar rings are labeled and colored orange (i.e., Y28, Y68, and Y110).

In addition to CBAs from higher plants, it should be noted that other lectins isolated from algae and Cyanobacteria (formerly classified as blue algae), which essentially recognize high-mannose glycans, have been identified as potential CBAs for pathogenic enveloped viruses (Table 3). In this respect, griffithsin, the Man-specific lectin purified from the red alga *Griffithsia* sp. [67], was investigated in detail as a relevant CBA for targeting the envelope protein from pathogenic enveloped viruses [68] because of its high affinity for oligomannosides [69] (Figure 10).

**Table 3:** List of algal and cyanobacterial lectins identified as carbohydrate-binding agents (CBAs) for envelope proteins from pathogenic enveloped viruses.

**Lectin**	**Algal Species**	**Carbohydrate-** **Binding** **Specificity**	**Targeted Envelope** **Protein**	**Virus**	**Ref.**
AML	*Amantia multifida*	Fetuin, mannan	hemagglutinin	IV	[52]
			E-glycoprotein	HSV	[52]
			gp120	HIV	[52]
BSL	*Bryothamnion seaforthii*	Fetuin, mucin	gp120	HIV	[52]
			E-glycoprotein	HSV	[52]
			hemagglutinin	IV	[52]
ESA-2	*Eucheuma serra*	Man	hemagglutinin	IV	[70]
GCL	*Grateloupia chiangii*	Man	hemagglutinin	IV	[71]
			E-glycoprotein	HSV	[71]
Griffithsin	*Griffithsia* sp.	Man	gp120	HIV	[72]
			E-glycoprotein	HCV	[73]
			S-protein	MERS-CoV	[74]
			S-protein	SARS-CoV	[68]
			S-protein	SARS-CoV-2	[75]
HML	*Hypnea musciformis*	Thyroglobulin, mucin	gp120	HIV	[52]
			hemagglutinin	IV	[52]
			E-glycoprotein	HSV	[51]
HTL-40	*Halimeda renschii*	Man	hemagglutinin	IV	[76]
KAA-2	*Kappaphycus alvarezii*	Man	hemagglutinin	IV	[77]
			gp120	HIV	[78]
MEL	*Meristiella echinocarpa*	Mannan	hemagglutinin	IV	[52]
			hemagglutinin	IV	[52]
SfL	*Solieria filiformis*	Mannan	E-glycoprotein	HSV	[52]
			gp120	HIV	[52]
			hemagglutinin	IV	[52]
BCA	*Boodlea coacta*	Man	E-glycoprotein	HSV	[79]
			hemagglutinin	IV	[79]
**Lectin**	**Cyanobacterial Species**	**Carbohydrate-** **Binding** **Specificity**	**Targeted Envelope** **Protein**	**Virus**	**Ref.**
MVN	*Microcystis aeruginosa*	Man	gp120	HIV	[80,81]
			E-glycoprotein	HSV	[81]
MVL	*Microcystis viridis*	Man	gp120	HIV	[82]
			E-glycoprotein	HCV	[83]
Cyanovirin-N	*Nostoc ellipsosporum*	Man	gp120	HIV	[49,84]
(CV-N)			E-glycoprotein 1,2	EBOV	[85,86]
			hemagglutinin	IV	[86]
			E-glycoprotein	HCV	[87]
			E-glycoprotein	HSV	[88]
			S-protein	SARS-CoV-2	[89]
	*Oscillatoria agardhii*	Man	gp120	HIV	[90]
OAA	*Scytonema varium*	Man	gp120	HIV	[91]
SVN	*Scytonema varium*		E-glycoprotein	DENV	[92]
			E-glycoprotein	EBOV	[93]

Although cyanobacterial lectins exhibit similar antiviral activities against pathogenic enveloped viruses, compared to other lectins from higher plants, they readily differ by the different fold and the smaller size of their structural scaffolds [93,94]. Moreover, cyanobacteria contain other small metabolites that could be used as valuable tools for combating enveloped viruses and, especially SARS-CoV-2 responsible for the COVID-19 pandemic [95].

## 5. How Can the Infectivity of Pathogenic Enveloped Viruses Be Affected by Lectins?

The glycan-mediated interaction of lectins with pathogenic enveloped viruses has a direct effect on virus infectivity, essentially by interfering with the recognition of their corresponding host cell receptors via different mechanisms. However, a dichotomy must be introduced between experiments performed in vitro on cultured cells and experiments achieved in vivo on animals.

Experimental studies performed in vitro, especially on HIV-infected cultured cells, have focused on higher plant lectins and cyanobacterial lectins [96]. Mannose-specific lectins have been recognized as the most efficient inhibitors of the HIV entry into the target cells by interacting with the glycan shield of gp120 and gp41, preventing their recognition by the CD4 receptors present on CD4+ T cells. Mannose-specific lectins of monocot plant species, such as *Cymbidium* hybrid (CHA), *Epipactis helleborine* (EHA), *Hippeastrum* hybrid (HHA), *Galanthus nivalis* (GNA), *Listera ovata* (LOA), and *Narcissus pseudonarcissus* (NPA), have been widely investigated in this respect by Balzarini and co-workers [53,54]. Other lectins with similar Man-binding specificity like BanLec from *Musa acuminata* [43] and Con A from *Canavalia ensiformis* [97] or different carbohydrate-binding specificities, such as the (GlcNAc)_n_-specific lectins Nictaba from *Nicotiana tabacum* [62], UDA from *Urtica dioica* [53], and WGA from *Triticum aestivum* [98], were also identified as potential inhibitors of the HIV entry into target cells in vitro and the syncytium formation resulting from the fusion of HIV-infected and HIV-uninfected CD4^+^ T lymphocytes [99,100].

A rather different situation can occur under in vivo conditions due to the multiplicity of cells susceptible to interacting with the virus. In addition, to block the entry of HIV particles into the cells and syncytium formation, plant lectins interfere with other mechanisms of virus infection and transmission, for example, by preventing the recognition of high-mannose glycans of gp120 by the DC-SIGN receptor of dendritic cells [101,102,103] or by blocking the transmission of DC-SIGN-captured virions to the CD4^+^ T lymphocytes [102]. In addition, as reported in [96], interaction with lectins of different carbohydrate-binding specificities can result in cytotoxic side effects on host cells, e.g., caspase-dependent apoptotic responses, due to the activation of different signaling pathways leading to apoptotic and necrotic responses that result from the recognition of surface-exposed *N*- and *O*-glycans by lectins.

The effects of plant lectins on other pathogenic enveloped viruses have been more scarcely investigated. Plant lectins were identified as blocking agents for the entry of IV [45,52,57], HSV [57], HCMV [47,53,54], EBOV [47,48], LASV [47], and the coronaviruses MERS-CoV [51], SARS-CoV [42,51,54,62,63,104], and SARS-CoV-2 [50,51,52,58,60,61,66] in their corresponding host cells. However, depending on the viruses, the envelope glycoprotein(s) targeted by plant lectins are extremely diverse as mentioned in Table 2. In this respect, an engineered banana lectin, BanLec, which has lost its mitogenic potential but retained its mannose-binding property, interacted with the envelope E-glycoprotein and inhibited both the entry and replication of Ebola virus in cell cultures [47,48]. The cyanobacterial lectin, cyanovirin-N (CV-N), also displayed similar inhibition towards Ebola virus [85]. Similarly, lentil lectin, LcA, inhibited the early steps of the host cell infection by SARS-CoV-2 and variants B.1.1.7 (α variant), B.1.351 (β variant), and P1 (γ variant), by blocking the recognition of their spike S protein by the ACE2 receptor [51].

The effects resulting from the binding of plant lectins on the different enveloped viruses also depend on the mechanisms of infection and transmission of these viruses, which may differ from those developed by HIV. In addition to preventing the entry and replication of Ebola viruses into the host cells in cell cultures, the engineered BanLec lectin, pre-administered to virus-infected mice, were highly protective against a lethal EBOV infection in vivo (~80% of mice protected) [48]. The engineered BanLec lectin was similarly efficient for protecting influenza virus-infected mice, by inhibiting the virus-endosome fusion occurring after the exogenous lectin has been internalized in the late endosomal/lysosomal compartment of the host cells [46]. Both plant lectins and griffithsin, the Man-specific lectin from the red alga *Griffithsia* sp., also inhibited the entry of SARS-CoV and MERS-CoV, respectively, in virus-infected cultured cells [42,74]. The Neu5Ac-specific *Maackia amurensis* lectin, MAL, inhibited the interaction of the SARS-CoV-2 S protein with the ACE2 receptor in cell cultures and decreased the expression of inflammatory mediators associated with COVID-19 disease progression [61].

The effects of plant lectins on coronaviruses were also investigated in virus-infected mice models. The (GlcNAc)_n_-specific lectin from the stinging nettle (*Urtica dioica*) (UDA), was shown to prevent virus entry and replication in a dose-dependent manner and reduced the virus infectivity significantly in a lethal SARS-CoV BALB/c mouse model [105]. Under in vivo conditions, the lectin from the hyacinth bean (*Lallab purpureus*), FRIL, neutralized H1N1 influenza by aggregating and trapping virions in the late endosomes of the host cells, thus preventing their nuclear internalization [60]. The lectin similarly neutralized SARS-CoV-2 by preventing viral protein production and cytotoxic effects on the host cells.

Algal and cyanobacterial Man-specific lectins, such as griffithsin and cyanovirin-N, also inhibited the entry of HIV and other enveloped viruses in the host cells in vitro and exerted in vivo cytotoxic effects very similar to those of plant lectins [74,75,76,77,78,79,80,81,82,83,84,85,86,87,88,89,90,91,92,93,94,95,96,97,98,99,100,101,102,103,105,106,107].

## 6. Biomedical Perspectives for Antiviral Lectins

Depending on their affinity towards surface-exposed glycans of enveloped viruses, plant lectins are considered as potential CBAs useful for combating viral infections, even though little evidence exists to date for their efficacy as relevant therapeutic tools [97,108,109,110,111,112]. Beyond their possible use as well-adapted tools for the diagnosis of viral infection, the therapeutic use of plant lectins as virus blockers faces practical and functional challenges which mainly concern (1) their large-scale production and (2) their unwanted immunomodulatory properties.

In most higher plants, lectins of different specificities that could be used as virus blockers occur as storage proteins in seeds and other vegetative organs such as tubers and rhizomes [113]. Man-specific two-chain (LcA, PsA, VfA, and LoL-I/II) and single-chain lectins (Con A, PHA, and SBA) from the Fabaceae are sequestered in the protein bodies of the cotyledonary cells in rather low amounts [114]. Accordingly, the extraction yield of legume seed lectins is rather low, in the range 50–80 mg/100 g (dry weight) seed [115]. However, the degree of purity of the extracted lectins is excellent since the introduction of affinity chromatography techniques using carbohydrate-immobilized columns. Different strategies have been developed recently to improve the extraction yield of griffithsin, the Man-specific lectin from the red alga *Griffithsia* sp., for the purpose of obtaining a large-scale production of the lectin able to supply the quantities of lectins needed for therapeutic applications [116,117,118,119,120,121]. These strategies are based on the continuous improvement of yields obtained from the high-level expression and extraction of griffithsin from transformed tobacco (*Nicotiana benthamiana*) leaves. In addition, griffithsin is easily purified and recovered from ensiled dried tobacco leaves, which allows for a low-cost production of lectin quickly adaptable to demand.

Most plant lectins consist of oligomeric structures built up from the non-covalent association of 15–20 kDa monomers in dimers and tetramers, more rarely in hexamers or octamers [31]. Depending on their structural organization, plant lectins usually exhibit a high degree of resistance to the degradation by trypsin-like proteases together with a pronounced capacity to trigger the synthesis of specific anti-lectin IgG. In this respect, IgG-binding epitopes have been identified on the molecular surface of Man-specific two-chain lectins from the Vicieae tribe [122,123], and monoclonal antibodies that specifically recognize lentil and *Lathyrus ochrus* lectins, were easily prepared [124,125]. Even Man-specific dietary lectins, such as BanLec from banana and ASA from garlic, have been reported to induce an immune response since specific anti-lectin IgG have been identified in the serum of banana and garlic consumers [126,127]. Through a specific interaction with the plant lectins associated to the enveloped viruses, these IgG could eventually neutralize the effects of lectins in the host cells. The Man-specific algal griffithsin and cyanobacterial lectins, such as cyanovirin-N and scytovirin, could overcome this challenge because of the small size of their composing monomers [128,129]. In addition to griffithsin, grifonin-1 (GRFN-1), an even smaller peptide of eighteen amino acids derived from griffithsin, has proven its efficacy as a blocking agent against HIV [130].

Initially recognized as potent mitogenic proteins [131,132,133], plant lectins have been known for a long time as non-specific immune-modulatory proteins that are susceptible to interaction with various cell surface glycoproteins/glycolipids and for interfering with various signaling pathways triggering cytotopathologic effects on the targeted cells. Although most plant lectins with antiviral activity activate different sets of T lymphocytes and, more scarcely, B lymphocytes, they also activate both the apoptotic and necrotic pathways in many other types of healthy and cancer cells [134,135,136,137,138,139,140,141,142,143]. The cellular activation mediated by plant lectins on healthy and transformed cells elicits the release of various chemokines and/or cytokines that are, in turn, susceptible to interfere with the cytokine stimulation associated with the viral infection, e.g., HIV infection [59]. However, plant lectins readily differ from each other by their capacity to elicit a cytokine production, some of them, such as PHA, Con A, and cyanovirin-N, being more active to induce the synthesis and release of activation markers [144,145], while Man-specific GNA-like lectins, such as GNA and HHA, were virtually incapable of triggering a relevant cytokine production [145,146]. Recently, a single-point mutation performed on an engineered banana lectin, BanLec, and an engineered Malaysian banana lectin, Malay BanLec, allowed to produce an active Man-binding lectin significantly devoid of mitogenic/cytotoxic activity [46,47]. If applicable for other Man-specific lectins, this point mutation approach would be an elegant way to attenuate or suppress the unwanted mitogenic/cytotoxic effects of lectins on target cells.

In spite of these limitations hampering the use of plant lectins as CBAs for combating pathogenic enveloped viruses, some ex vivo applications of plant lectins have been successfully developed. An important decrease in the plasma load with Ebola virus was achieved by extracorporeal affinity plasmapheresis of the contaminated blood through a GNA-immobilized matrix [147]. Recently, *ex-vivo* plasmapheresis on a Man-specific lectin-immobilized column of blood spoiled by the MERS-CoV and the Marburg viruses has proven its efficacity to purge the blood samples from virus particles [148]. Although essentially theoretical, the risk of a possible transfusion transmission of SARS-CoV-2 with spoiled blood samples should be avoided by a simple lectin plasmapheresis step of the suspected blood samples [149,150]. Another ex vivo application of plant lectins has been proposed on the web (Pittsburgh University, 2020) in the form of a nasal spray of griffithsin that could be used to prevent the infection by SARS-CoV-2 and other pathogenic enveloped viruses, e.g., in immune-compromised people. A lectin spray could also be used to detect the enveloped viruses on various domestic surfaces, such as doorknobs, handrails, computers, and cooking tools, under UV illumination after labeling with specific anti-lectin antibodies coupled to a fluorochrome.

## 7. Bioinformatics

Atomic coordinates of fusion proteins, B glycoproteins, and E glycoproteins were taken from the Protein Data Bank (PDB): 7JPH (EBOV) [5], 6Y5G (IV) [6], 5CXF (HCMV) [7], 2GUM (HSV) [8], 4TVP (HIV) [9], 5VK2 (LASV) [10], 5W9H (MERS-CoV) [11], 6ACD (SARS-CoV) [12], 6VXX (SARS-CoV-2) [13], 3N40 (CHIV) [14], 1UZG (DENV) [15], and 7BUB (ZIV) [16].

The molecular surface of the lectins and envelope glycoproteins from pathogenic enveloped viruses were calculated and displayed with Chimera [151] and Chimera-X [152]. Assuming that putative *N*-glycosylation sites, NXT/S, of envelope glycoproteins are actually glycosylated, a classic *N*-glycan chain corresponding to the trimannoside core Man_3_GlcNAc_2_, was modeled using the GlyProt server (http://www.glycosciences.de/modeling/glyprot/php/main.php) (accessed on 22 December 2021) [153] and represented in CPK on the molecular surface of the envelope glycoproteins.

The illustrations of the high-mannose *N*-glycans, complex *N*-glycans, hybrid *N*-glycans, and *O*-glycans were built and represented with the DrawGlycan SNFG package for Mac [154]. Colored symbols were used to represent Fuc (red triangle), Gal (yellow circle), Glc (blue circle), GalNAc (yellow square), GlcNAc (blue square), Man (green circle), and sialic acid/Neu5Ac (purple diamond).

## 8. Discussion

The glycan shield covering pathogenic enveloped viruses plays a role not only in the protection of viruses but also in various important mechanisms insuring the entry and replication of viruses in the host cells [30]. Thus, the recognition of the glycan shield by plant lectins provides a way to fight viral infection and, especially, the SARS-CoV-2 infection, by competing with the spike-mediated attachment of viral particles to the host cell virus receptors. However, due to the extreme diversity of *N*-glycan types covering enveloped viruses, especially beta-coronaviruses [15], plant lectins with different carbohydrate-binding specificities should be tested for this purpose. From experiments performed under in vitro and in vivo conditions, it follows that plant, algal, and cyanobacterial lectins with different carbohydrate-binding specificities represent well-adapted CBAs for blocking the entry of pathogenic enveloped viruses into the host cells. In this respect, Man-specific lectins of the Vicieae tribe, which specifically recognize the α1,6-fucosylated Man_3_GlcNAc_2_ core of *N*-glycans of the complex- and hybrid-type, are particularly relevant as glycan probes for the beta-coronaviruses MERS-CoV, SARS-CoV, and SARS-CoV-2 [51,106]. However, Man-specific lectins are not considered as replication blockers for coronaviruses, since they do not interfere with the coronavirus replication within the cell.

Despite the accumulating evidences that plant lectins and, especially, Man-specific plant lectins, could be used as tools for preventing infection by pathogenic enveloped virus, in particular SARS-CoV-2 responsible for the COVID-19 pandemic, some unwanted characteristics of plant lectins make these molecules difficult to use for a therapeutic purpose. Due to the fact of their high molecular size, which favors the synthesis of anti-lectin antibodies, and their mitogenic/cytotoxic properties, which interfere with the cytokine response of infected individuals, their use is limited to external treatments. However, the promising results obtained with a single-mutated engineered banana lectin, BanLec, which retains its carbohydrate-binding ability but loses its mitogenic property [46,47], could pave the way for the forthcoming production of innocuous mutated plant lectins available for a therapeutic use.

In addition to lectins, other small molecules could be used as blockers for the SARS-CoV-2/ACE2 interaction. Recently, some small molecular drugs, including dyes, glycosides, tannins, and immunosuppressors, were characterized as either spike or ACE2 binders susceptible to blocking the attachment of the SARS-CoV-2 spikes to ACE2 by interfering with the ligand and/or the receptor surface [155]. Depending on the *N*-glycan types that are linked to the receptor DPP4 for MERS-CoV and SARS-CoV viruses [156] and ACE2 for SARS-CoV-2 virus [157], plant lectins with Man-binding activity could interfere with the capture of the beta-coronavirus spikes by their corresponding host cell receptors. This dual activity of plant lectins towards the glycans of spikes and their receptors is of paramount importance for reinforcing the antiviral properties of plant lectins against pathogenic beta-coronaviruses.

## Data Availability

Not applicable.
